# DNA methylation profiling for molecular classification of neuroblastoma

**DOI:** 10.1186/s13148-025-01936-7

**Published:** 2025-07-27

**Authors:** Maja Löfgren, Anna Djos, Shiva Rezaei, Medha Suman, Per Kogner, Tommy Martinsson, Susanne Fransson, Helena Carén

**Affiliations:** 1 Department of Medical Biochemistry and Cell Biology, Sahlgrenska Center for Cancer Research, Institute of Biomedicine, Sahlgrenska Academy, University of Gothenburg, Gothenburg, Sweden; 2 Department of Laboratory Medicine, Institute of Biomedicine, Sahlgrenska Academy, University of Gothenburg, Gothenburg, Sweden; 3 Childhood Cancer Research Unit, Women’s, and Children’s Health, Karolinska Institutet, Stockholm, Sweden

## Abstract

Neuroblastoma is a heterogeneous disease where patient stratification is critical for prognosis and treatment decisions and where it recently has been suggested that the presence of telomere maintenance mechanisms (TMM) should be considered in risk stratification. We investigated the utility of DNA methylation-based classification for neuroblastoma diagnostics by analysing 303 tumours samples from two cohorts. We show that of the total number of cases, an average of 90% of the samples classified as neuroblastoma, while 66% also achieved confident classification into the three NB subclasses: “MYCN-type”, “ALT/TERT TMM positive” and “TMM negative”. The tumours classified as MYCN-type showed genomic amplification of *MYCN* (MNA); however, some MYCN-type cases lacked evident MNA, suggesting that epigenetic states might be influenced by other factors such as activating *ALK* mutations. Survival analysis indicated similar poor survival probabilities for patients classified as TMM positive or MYCN type, distinct from the inferior survival of TMM-negative cases. All cases, with complementary genomic data available, associated with TMM positivity also presented features associated with telomere lengthening mechanisms, including *TERT* or *ATRX* alteration. However, some tumours positive for these features, especially *TERT* rearrangement, classified as MYCN type rather than TMM positive, indicating that MNA and other mechanisms introduce a methylation pattern that supersede or overlap with pattern imposed by *TERT*. Chromosomal copy number alterations (CNAs) characteristic of methylation subclasses were identified, including 1p deletion and 17q gain in MYCN type and combinations of 11q loss, 3p loss, 7q gain, and 17q gain in TMM-positive cases, highlighting the potential of the methylation arrays to replace SNP arrays for prognostic genomic assessments. Our study demonstrates that DNA methylation-based classification stratifies neuroblastoma into clinically relevant subgroups, aiding diagnostic and prognostic decisions, although discrepancy between genomic features and methylation classification does occur. The interplay between genomic alterations and methylation patterns could give clues into the discrepancy and underscores the complexity of neuroblastoma biology and the need for further research and validation of clinical outcomes of the patients in the respective subclasses.

**Supplementary Information:**

The online version contains supplementary material available at 10.1186/s13148-025-01936-7.

## Introduction

Neuroblastoma (NB) is a paediatric tumour derived from immature cells of the peripheral sympathetic nervous system. NB have highly heterogeneous clinical and biological behaviour, ranging from high-grade tumours with high degree of adverse outcome of patients to tumours that can spontaneously regress. Despite intense multimodal treatment, the overall mortality remains high at about 20–35%, and more than 50% of the patients with high-risk NB will eventually succumb to the disease [1]. Risk assessment of NB patients is currently based on age at diagnosis, stage of disease and presence of genomic alterations such as *MYCN* amplification (MNA) and 11q-deletion (11q-del) [2]. MNA and 11q-del are two almost mutually exclusive events in NB and defines two biologically distinct groups that differ in age at diagnosis and number of segmental copy number alterations [3]. Beside MNA and 11q-del, other frequently occurring alterations in NB include deletion of chromosomal arms 1p, 3p and 9p, and gain of 1q, 2p, 7q and 17q [3]. In addition, other genomic aberrations have been reported to target genes in cell cycle regulation, neural function or telomere maintenance, i.e. *TERT* (encoding the catalytic subunit of telomerase) and *ATRX* (encoding a chromatin remodelling protein).

The telomeres are essential structures that cap the chromosomes to retain DNA integrity while also providing an inherent replicative limitation due to telomere erosion. When shortened telomeres reach a critical threshold, normal cells will enter senescence or apoptosis. However, a hallmark of most malignant tumours is the capacity to undergo unlimited rounds of cell divisions, which requires presence of a telomere maintenance mechanism (TMM). This is commonly achieved through reactivation of *TERT* or the alternative lengthening of telomeres (ALT) pathway. ALT is a telomerase independent mechanism that instead hijacks the homologous recombination pathways to elongate the telomeres. ALT is associated with accumulation of extrachromosomal telomeric circles (c-circles), long telomeres of heterogeneous lengths and mutations in *ATRX*, or more rarely *DAXX* [4, 5]. In NB tumours with MNA, telomere maintenance is commonly achieved through re-expression of *TERT*, which is promoted by direct binding of MYCN to the E-box consensus sites within the *TERT* promoter region [6]. In non-MNA tumours, it has been shown that telomere maintenance is frequently achieved either through (i) genomic rearrangements that place *TERT* under the control of strong enhancer elements [7] or (ii) ALT, which is associated with *ATRX* alterations in 55–60% of all ALT-positive cases [8, 9]. Evaluation of telomere function in large cohorts has shown that presence of TMM in NB is highly connected with very poor prognosis regardless of risk group or stage. This has led to a suggested model of NB pathogenesis where presence of TMM is key factor in risk assessment [10].

DNA methylation is an epigenetic mechanism that regulates gene expression without altering the underlying nucleotide sequence and is crucial for normal cellular processes such as differentiation, X-chromosome inactivation, genomic imprinting and gene suppression. In cancer, DNA methylation patterns reflect both the cell type of origin and acquired changes during tumour formation. This phenomenon has been leveraged for various paediatric CNS tumour entities where methylation-based classification has emerged as an important molecular platform. This allows classification with improved diagnostic accuracy and patient risk stratification in comparison with the standard of care histopathological analysis and any single molecular tests and has already entered clinical diagnostics in several countries [11–13]. Several attempts to perform risk stratification based on epigenetic profiling have been made also for NB. We and others have shown there is a prognostic value of broad DNA methylation analysis and, that it is indeed possible to separate different subgroups of NB based on epigenetic features [14–19]. Although these studies provide valuable insights of the methylome in relation to clinical outcome, none of these are specifically addressing TMM. However, in an updated version of the widely employed brain tumour classifier, the Molecular Neuropathology (MNP) (www.epignostix.com), the methylation class “neuroblastoma” with three different subclasses was added. The included subclasses are “MYCN type”, “Telomere Maintenance Mechanism (TMM) negative”, and “ALT/TERT TMM positive”.

To our knowledge, no evaluation on the performance of the MNP classifier for neuroblastoma samples has been published yet. Thus, we analysed two separate cohorts of a total of 303 NB cases using the available MNP classifier and correlated methylation classification with genetic alterations of the tumours and survival of the patients to assess its diagnostic and prognostic potential.

## Results

We used a local cohort of 90 neuroblastoma subjects and the public available DNA methylation array dataset of 213 neuroblastoma samples in TARGET, Table 1.

**Table 1:** Patient characteristics of the local and of the TARGET neuroblastoma cohort

Characteristics	Local cohort	TARGET cohort
Number of patients	90	213
Gender (n, %)		
Male	36 (40%)	120 (56%)
Female	54 (60%)	93 (43%)
Age		
Age at diagnosis: Median (range; years)	1.8 (0–18.5)	2.8 (0–20.9)
Patients < 1.5y: n (%)	35 (38)	46 (21)
Patient ≥ 1.5y: n (%)	55 (61)	167 (78)
MYCN status: n (%)		
Amplified	31 (34)	50 (23)
Not amplified	58 (64)	161 (76)
Unknown	1 (1)	2 (1)
INSS: n (%)		
Stage 1	1 (1)	15 (7)
Stage 2	8 (9)	1 (0)
Stage 3	12 (13)	6 (3)
Stage 4	39 (43)	167 (78)
Stage 4 s	3 (3)	24 (11)
Unknown	27 (30)	–
Year of diagnosis: Median (range)	2008 (1986–2023)	2007 (1998–2011)

### DNA methylation-based classification

**Fig. 1:**
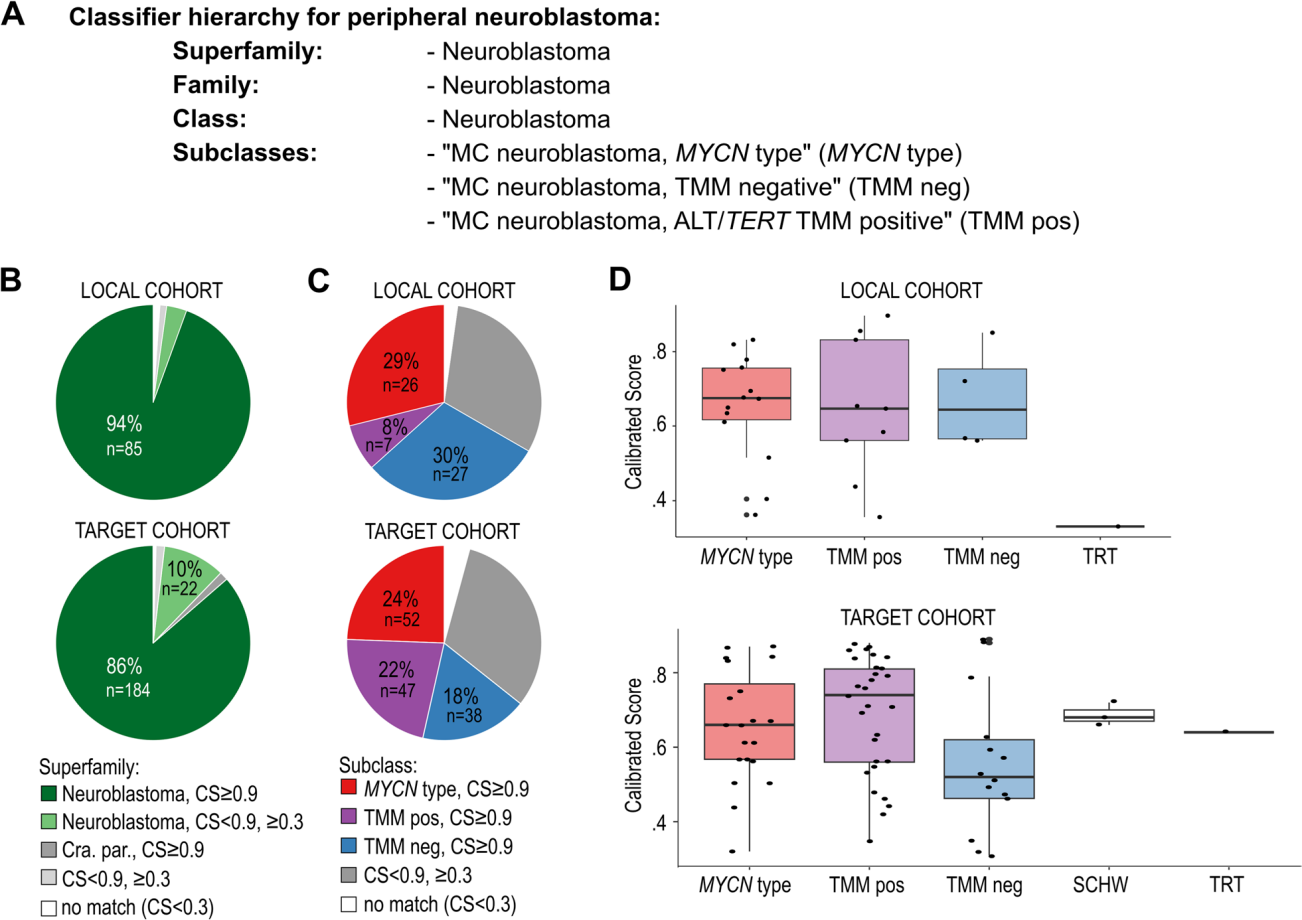
Classification of NB tumours using the MNP Classifier. **A** The hierarchy tree of classes for peripheral NBs in the MNP classifier v12.5. **B**, **C** Classification of the local (*n* = 90) and the TARGET cohort (*n* = 213) at the Superfamily (**B**), and at the Subclass level (**C**), grouped using the recommended Calibrated Score (CS) cut off level ≥ 0.9. **D** Subclass association of tumours that received a CS < 0.9, and ≥ 0.3 in the local cohort (top panel, *n* = 28), and in the TARGET cohort (lower panel; *n* = 67). CS, Calibrated Score; Cra. Par., the superfamily “Cranial and Paraspinal Nerve Tumours”; TRT, the molecular class “Teratoma (novel)”; SCHW, the molecular class “Schwannoma”

We explored the ability of the DNA methylation-based classifier MNP v12.5 to classify NB samples. The classifier has a hierarchical structure, and each classification is associated with a calibrated score (CS). The levels ranging from Superfamily to Molecular Class (MC), at the Subclass level, are harmonized with other disease entities in the classifier. In v12.5, and in the later version 12.8 of the classifier, the arm of peripheral neuroblastoma has the same calibrated score for the class “neuroblastoma” at the levels of Superfamily – Class, while the Subclass has three nodes: “MC Neuroblastoma, MYCN type”, “MC neuroblastoma, TMM negative” and “MC neuroblastoma, ALT/TERT TMM positive” (Fig. 1A). A sample is classified when the CS is ≥ 0.9, and we use the term associated for when the CS for a suggested class is < 0.9 but ≥ 0.3, while a sample with CS < 0.3 is seen as no match and thereby unclassifiable. We applied the classifier to the local and the TARGET dataset of methylation arrays and collected the output.

### Classification on superfamily level

We first investigated to which Superfamily the samples classified. In the local cohort, 94% (85 cases) classified into neuroblastoma (Fig. 1B), while three samples (3%) were associated (CS 0.5–0.89). One sample was associated with the Superfamily “Germ cell tumours” but with low score (CS < 0.4). Thus, no sample classified with confident CS outside of the expected diagnosis in this tumour set. In the TARGET dataset, 86% (*n* = 184) of samples classified into the Superfamily neuroblastoma, while 22 samples (10%) were associated (CS 0.33–0.89; Fig. 1B). Three samples classified into the Superfamily “Cranial and Paraspinal Nerve Tumours”. One sample associated with the Superfamily “Germ Cell Tumours” (CS 0.66), and two samples associated with low scores with “Cranial and Paraspinal Nerve Tumours” (CS 0.45) and “Medulloblastoma” (CS 0.36), respectively. In both cohorts, one sample each was unclassifiable (CS < 0.3). Both these samples were boys less than 5 years old, with disease classified as INSS stage 4, and without *MYCN* amplification. The case from the local cohort had a *c-MYC* amplification and died within a year of disease discovery, while the TARGET case had an unfavourable histology with the diagnostic category “Ganglioneuroblastoma, nodular”, but the patient was alive after > 10 years of follow-up (last follow-up).

### Classification on subclass level

We next explored the classification at the Subclass level. In the local cohort, 60 samples (60/90; 67%) classified into neuroblastoma subclasses: 27 samples (27/90; 30%) into TMM negative, 26 samples (26/90; 29%) into *MYCN* type, and seven samples (7/90; 8%) into “ALT/*TERT* TMM positive” subclass (Fig. 1C). Out of the remaining samples, 14 samples were associated with “*MYCN* type”, nine with TMM positive, and four with the TMM negative subclass (Fig. 1D, upper panel). One sample was weakly associated with MC “Teratoma (novel)” (CS < 0.4). This sample was *MYCN* amplified and associated with Superfamily “Germ cell tumours” (CS < 0.4). Two samples had no match (CS < 0.3). These were samples without *MYCN* amplifications, but with *c-MYC* amplification, and amplifications on chromosome 12, respectively.

In the TARGET cohort, 137 samples (64%) obtained a CS ≥ 0.9. The most common classification was to the class “*MYCN* type” (52/213; 24%), followed by “ALT/*TERT* TMM positive” (47/213; 22%,) and “TMM negative” (38/213; 18%) (Fig. 1C). No sample classified outside of NB subclasses. Most of the remaining samples (63 samples) were associated with a NB subclass with varying CS (Fig. 1D). Three samples associated with “MC Schwannoma” (CS 0.66—0.72). These three samples were from female subjects between 18 months and 5 years of age, INSS Stage 4, COG high risk, without amplified *MYCN* (unknown for one sample), and with diploid genomes and classified into the Superfamily “Cranial and Paraspinal Nerve Tumours” (CS > 0.9). In addition, one sample, from a male subject taken a few days after birth, received a CS of 0.64 for “MC Teratoma (novel)”. This case was reported as an INSS Stage 1 and COG low risk, hyperdiploid, lacking *MYCN* amplification, with undifferentiated or poorly differentiated histology, and was associated with the Superfamily “Germ Cell Tumours” (CS of 0.66). Nine samples had no match (CS < 0.3) at subclass level.

### Evaluation of the ALT/TERT TMM positive class

**Fig. 2:**
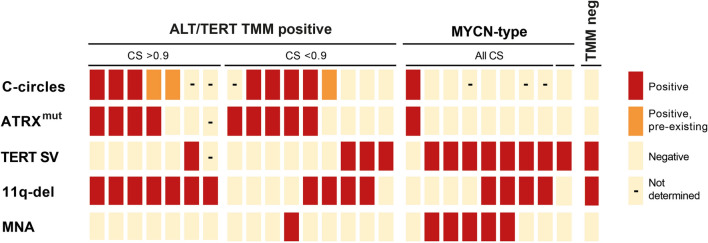
Overview of TMM positive classified, or associated, tumours, and of additional tumours with genomic features associated with telomere maintenance mechanism (TMM) in the local cohort. The group of the cases with genomic alterations associated with TMM, that did not classify as such, consisted of nine samples that classified into (*n* = 3) or associated with (CS 0.4–0.77, *n* = 6) the MYCN type whereof five cases did present MNA. One *TERT* positive sample associated with TMM neg (CS 0.72). Red, has feature; orange, positive for c-circles but pre-existing prior amplification; and black line indicates where data are not available. SV, structural variant; CS, classification score

Next, we investigated the presence of genomic support for ALT/*TERT* TMM classification among the samples of the local cohort that was either classified into (n = 7) or associated with (*n* = 9) this methylation group. For this, we used previously published data on *TERT* or *ATRX*-associated aberrations detected with SNP-microarray and/or whole genome sequencing and presence of c-circle as indication of ALT [5]. Out of the seven samples classified as ALT/*TERT* TMM positive, high-quality data were available for six cases (Fig. 2). Among these six, a *TERT* aberration was detected in one case, while *ATRX* alterations were present in four. All four *ATRX* positive cases showed presence of c-circles including one case with a missense mutation which showed pre-existing (i.e. detectable already prior amplification) single-stranded telomeric G-strand together with c-circles, designated as ambiguous presence of c-circles. One case which classified into the ALT/*TERT* TMM group lacked detectable alterations of *TERT* or *ATRX* but showed extensive telomeres, also a feature of ALT, together with ambiguous presence of c-circles. For the nine samples associated with the ALT/*TERT* TMM positive NB subclass, eight samples had genetic data clearly supporting the association, either by *TERT* rearrangements (*n* = 3), or *ATRX* gene alterations (*n* = 5). The remaining sample showed extensive telomeres together with ambiguous presence of C-circles in the absence of detectable *ATRX* aberration. Thus, supporting evidence for ALT/*TERT* TMM positive classification was present in all cases for which data were available (Fig. 2). One of the samples presented with *MYCN* amplification together with an *ATRX* alteration and presence of C-circles received classification scores of 0.56 and 0.39 for ALT/*TERT* TMM positive and *MYCN* type group, respectively. Among the ten sample with genomic changes in the *ATRX* gene, only one sample did not classify or associate as ALT/*TERT* TMM positive (Fig. 2). This sample associated with *MYCN* type (CS 0.65), despite absence of MNA. The subclass with the next highest CS was ALT/*TERT* TMM positive, but as unclassifiable (CS < 0.3).

*TERT* structural variations, which are associated with telomere retention [7, 10], were identified in 13 samples of the local cohort (Fig. 2). One of these samples classified as TMM positive (described above), and three of these classified as *MYCN* type together with genomic data supporting MNA. The nine samples associated with a MC NB Subclass with CS < 0.9 were distributed as follows: ALT/*TERT* TMM positive (*n* = 3), *MYCN* type, (*n* = 5, whereof two with MNA), and TMM negative (*n* = 1). As previously reported [5], deletion of chromosome 11q is frequently co-occurring with different TMMs. All seven cases classified as ALT/*TERT* TMM positive had 11q-deletion and four of the eleven that were associated (Fig. 2). Among the nine cases with *TERT* structural variations that did not classify or associate with the ALT/*TERT* TMM positive class, 11q deletion was present in five cases whereof co-occurrence with MNA in two instances.

For the TARGET cohort, similar genomic analyses were not available for validation of supporting evidence of the derived methylation class. However, in the TARGET cohort, the ALT/*TERT* TMM positive classified samples were COG high risk, INSS stage 4 and presented with unfavourable histology (Supplemental Fig. 1). There was also an overrepresentation of samples that were diagnosed as “Ganglioneuroblastoma, nodular” (Supplemental Fig. 1). On group level, the patients that classified as ALT/*TERT* TMM positive were older than those that classified as TMM negative or TMM positive (*P* value < 0.001 for both comparisons, Supplemental Fig. 2A), as expected as 11q-deleted neuroblastoma patients, which are enriched in the TMM positive class, are older at time of diagnosis than other genomic subgroups [3].

### Evaluation of the MYCN type class

There were 31 samples with MNA in the local cohort, assessed by SNP-microarray, whereof 22 (71%) classified to and six (19%) associated with the *MYCN* type methylation class. Among the three cases with MNA that did not receive a CS ≥ 0.9 for the *MYCN* type, one was associated with ALT/TMM positive (as discussed above), one sample was associated with the “MC Teratoma (novel)” class with a low CS (0.33), and one classified as TMM negative. The latter was a tumour from a 11-month-old female who presented with stage 4 progressive NB with co-amplification of *MYCN* and *ALK* that led to death within 1 year from diagnosis.

Twelve samples lacking MNA still classified into or associated with the *MYCN* type group (*n* = 4 and *n* = 8 respectively). The four samples with CS ≥ 0.9 had genomic profiles hallmarked by either 11q deletion (*n* = 2), or 17 gain without co-occurring MNA or 11q deletion (Supplemental Table 1). Among the cases that were associated with the *MYCN* type class, the CS for MNA samples was indeed higher than for the samples without MNA (*P* value 0.03; range CS 0.63–0.83, and 0.36–0.76, respectively). One of the cases associated with the MYCN type (CS 0.65) despite presence of an inversion with breakpoint within *ATRX*. This case also presents a R1275Q *ALK* mutation (data not shown).

In the TARGET cohort, 50 samples had MNA whereof 88% (44/50) classified as *MYCN* type, and two samples associated with the *MYCN* type class (CS > 0.6, for both). One case with evident presence of MNA unexpectedly classified as TMM negative (score 0.99). This sample was derived from a 6-year-old with a 4S NB and high risk, and despite a relapse event, the patient was still alive at last follow-up (> 7 years). The three remaining *MYCN*-amplified samples either associated with the TMM negative class (CS 0.53) or were unclassifiable (*n* = 2). Among the samples lacking genomic amplification of *MYCN*, 5% (8/161) and 11% (18/161), respectively, yet classified or associated with the *MYCN* type group.

**Fig. 3:**
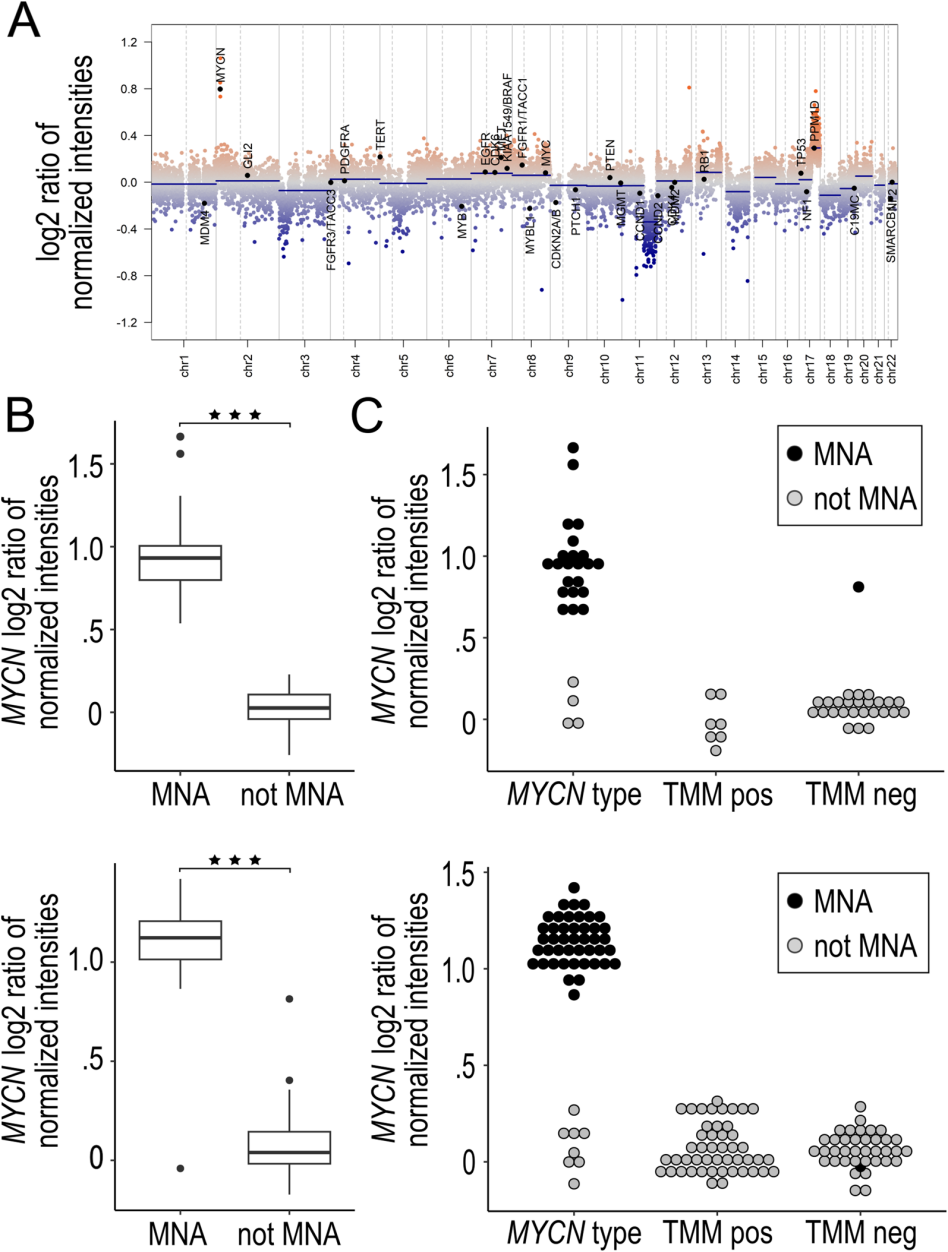
Evaluation of copy number alterations (CNA) estimated from DNA methylation data. **A** Exemplified genome plot with estimated CNA from R package conumee 2.0 of a tumour that shows signs of MYCN amplification. **B** Conumee-estimated relative *MYCN* copy number estimations are higher in *MYCN*-amplified (MNA), than in not MNA tumours, in both the local (top panel) and in the TARGET (lower panel) cohort. **C.** Tumours with methylation-based molecular classes of CS ≥ 0.9 are plotted against conumee-estimated relative MYCN amplification, in the local cohort (top panel) and in the TARGET cohort (lower panel). In both cohorts, MYCN status was lacking for one tumour, respectively. MNA, *MYCN*-amplified; ***, *P* value < 0.001

We next investigated if copy number alterations of *MYCN* inferred from the DNA methylation array data agreed with the methylation-based classification, and with SNP-microarray derived *MYCN* status. For this purpose, we used the conumee 2.0 package that estimate CNAs over the genome, as exemplified in Fig. 3a. The estimation of MNA was collected for each sample. This showed as expected that the methylation derived estimates for *MYCN* amplification in samples with MNA (as assessed by SNP-array) were significantly higher than the estimates for samples without MNA in both the local and in the TARGET cohort (Fig. 3b; *P* value < 0.001, Mann–Whitney U Test). All but one sample with MNA had a conumee-based *MYCN* estimate above 0.5 (Fig. 3). There was one sample without reported presence of MNA in the available genomic metadata that yet presented with a high *MYCN* amplification estimate as inferred from the methylation array. This was a three-year-old patient, from the TARGET cohort, with stage 4 disease that relapsed and led to death 4 years after diagnosis. The primary tumours sample obtained a CS of 0.81 for the TMM positive class. Unfortunately, no methylation data were available from the tumours at time of relapse. The *MYCN*-amplified sample that classified into the TMM negative class in the local cohort had a high conumee-based *MYCN* estimate (Fig. 3C, also discussed above), supporting the MNA status.

The samples that confidently classified as *MYCN* type without MNA, all indeed had low values with conumee-based *MYCN* estimation, both in the local and in the TARGET cohort (all values < 0.3; Fig. 2B). For samples that received a CS < 0.9, the conumee-based MYCN estimate was > 0.5 for eight MNA samples in the local cohort and for three MNA samples in the TARGET cohort (Supplemental Fig. 3).

### Evaluation of the TMM negative class

In the local dataset, 82% of the samples that were classified to the TMM negative class (CS > = 0.9) had a genomic profile, assessed from previous SNP microarray analysis [3], in agreement with lower risk profile groups; “numerical only” or “other segmental” (*n* = 17, and *n* = 5, respectively, Supplemental Table 1). The remaining five samples had *MYCN* amplification (n = 1), or 17q gain (with absence of *MYCN* amplification and 11q deletion, *n* = 4; Supplemental Table 1).

In the TARGET dataset, samples that classified to the TMM negative group were younger (median age 0.2 years, with interquartile range, IQR, 0.5 years) than samples that classified into the *MYCN* type (age median 2.7, IQR 2.3 years), and into the ALT/*TERT* TMM positive (age median 4.0 years, IQR 2.4 years) classes *(P* < 0.001, for both tests; Supplemental Fig. 2A). The samples classified as TMM negative had the highest proportion of INSS stage 1, 2b and 4S (Supplementary Fig. 1). For stage 4S, 22 samples classified into TMM negative, while the remaining two were associated with the TMM negative class (CS of 0.32, and 0.63, respectively). An additional four samples would be anticipated to classify into the TMM negative class, based on being INSS stage 1 and having a favourable outcome. Two were associated with this class (CS 0.40 and 0.88), and the remaining samples associated with the ALT/*TERT* TMM positive class (CS 0.71) and with “MC Teratoma (novel)” (CS 0.64). There were one single INSS 2b sample in the TARGET cohort that did not classify as TMM negative. This sample had a CS of 0.88 for the association with the *MYCN* type class, despite lacking MNA, and is further discussed below at the section of samples with multiple samplings.

### Genomic copy number aberrations associated with the molecular subclasses

**Fig. 4:**
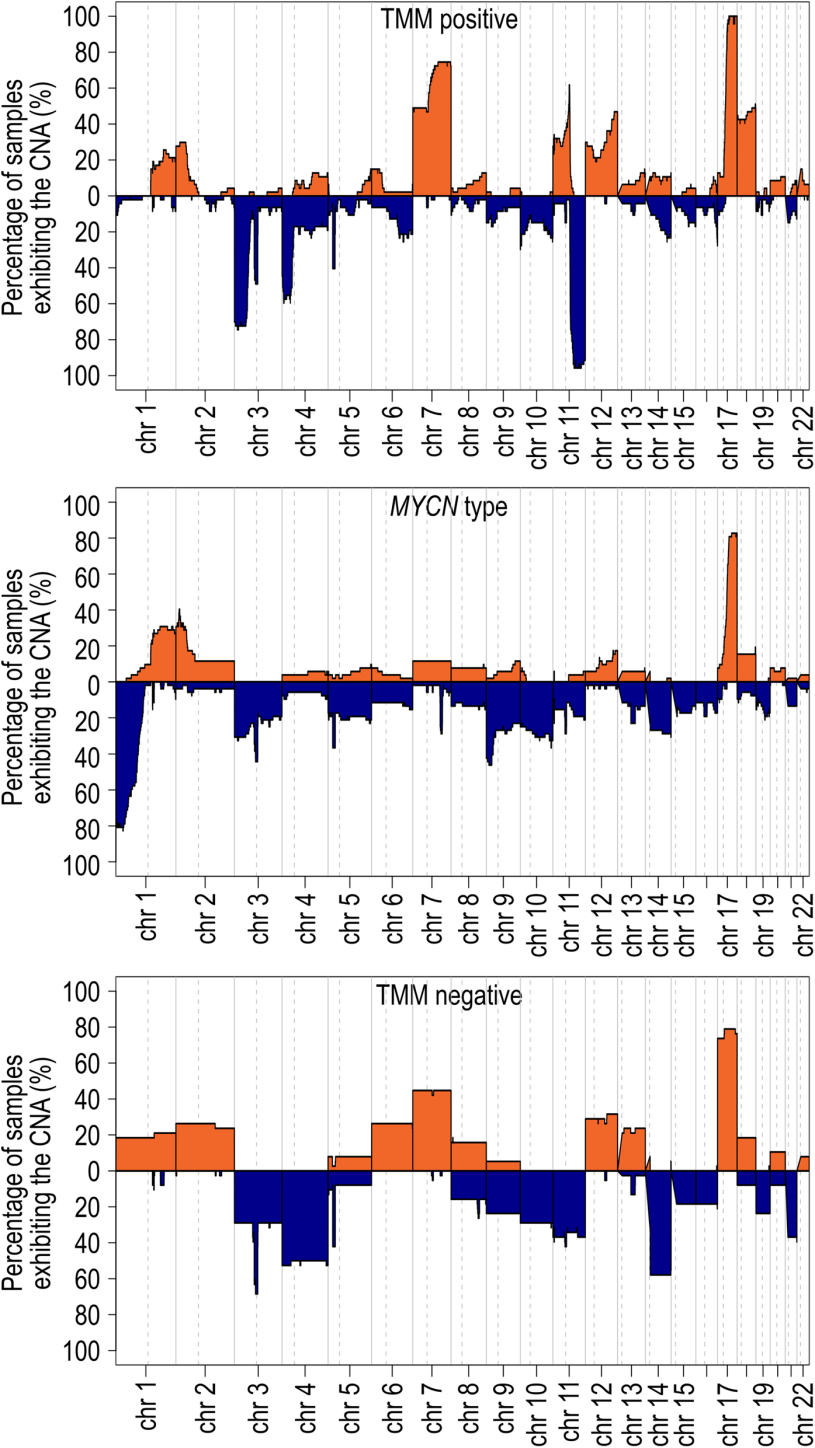
Summary copy number prediction plots of neuroblastoma subclasses in the TARGET cohort. Percentage distribution of copy number alterations in samples that classified into TMM positive (n = 47), MYCN type (*n* = 52) and TMM negative (*n* = 38) with CS ≥ 0.9 in TARGET cohort

Next, we searched for representative patterns of CNAs for the subclasses. For this purpose, the function summary plots of the conumee 2.0 package were used to find percentage of samples that shows CNA for binned regions over the genome. The analysis was done for each of the molecular subclasses and the respective cohorts. This analysis confirmed that the majority of TMM positive classified samples had 11q deletion (> 90% in both cohorts; Fig. 4, and Supplementary Fig. 4). Gain of chromosome 17 (TMM negative samples) or 17q (*MYCN* type, or TMM positive samples) was present in more than 60% of samples in all subtypes, in both cohorts (Fig. 4, and Supplement Fig. 4). Other frequent CNAs that were present in both the local and in the TARGET cohort were 1p loss (*MYCN* type samples, > 60%), 3p loss (TMM positive samples, > 60%) and gain of chromosome 7 or 7q-gain (TMM positive samples, > 70%; TMM negative samples > 40%). For the samples that classified into *MYCN* type, the summary plot showed an excess of 2p including the *MYCN* locus in 30–50% of samples, thereby not truly capturing the extent of focal *MYCN* amplification, which is a limitation of this visualization. For the TMM negative samples, the summary plot had a more boxed appearance, indicating enrichment of cases with numerical aberrations involving whole chromosomal gains and losses.

### Methylation classification and association with overall survival

**Fig. 5:**
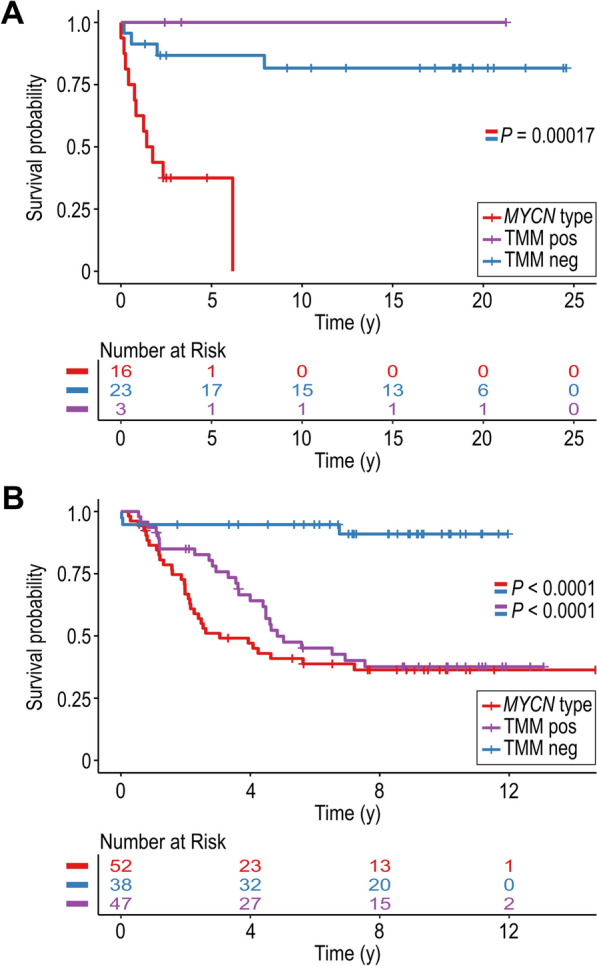
Overall survival probability for primary tumours that classify at CS ≥ 0.9 using the methylation-based classification. **A** Kaplan–Meier plot from the survminer package shows difference in survival probability between the classes MYCN type, and TMM NEG, in the local cohort (*P* = 0.00017). **B.** Kaplan–Meier plot from the survminer package shows differences in survival probability between the classes TMM NEG versus both MYCN type (*P* < 0.0001), and TMM POS (*P* < 0.0001), in the TARGET cohort

Samples that classified into *MYCN* type, or into ALT/*TERT* TMM positive were anticipated to be associated with poor outcome, compared to TMM negative samples. In the local cohort, extended survival data were available for patients represented by primary tumours for 42 cases. The patients with tumours classified as TMM negative had a significant better overall survival than the *MYCN* type cases (*P* 0.00017; Fig. 5A). There were only three subjects in the ALT/*TERT* TMM positive class, with no death occurrences. To further evaluate the classification prediction, we analysed the risk prediction of the samples that classified and those that were associated. For this analysis, we filtered away the weakest associations by applying a CS limit of ≥ 0.5, which increased the dataset to 56 samples, out of which 6 cases were associated with, or classified into ALT/*TERT* TMM positive. While the overall *P* value was < 0.0001, the pairwise class tests were only significant for the TMM negative class *vs* the *MYCN* type class (*P* < 0.0001; Supplementary Fig. 5A).

The Kaplan–Meier plot of the 137 classified samples in the TARGET cohort is shown in Fig. 5. The overall survival was indeed longer in the TMM negative cases compared with the *MYCN* type cases (*P* < 0.0001), and to the ALT/*TERT* TMM positive samples (*P* < 0.0001). In this larger cohort, the overall survival did not differ between the *MYCN* type and the ALT/*TERT* TMM positive cases (*P* > 0.2), despite a steeper slope for the MYCN group (Fig. 5B). Similar results were obtained for the NB subclasses when using the less stringent limit of CS ≥ 0.5 (Supplementary Fig. 5B).

Next, we explored the classification prediction within each neuroblastoma MC subclass between samples that classified *vs* samples that were associated in the larger TARGET cohort. There was no significant difference in survival prediction between classified and associated samples of the *MYCN* type or the TMM positive subclasses (Supplementary Fig. 6), respectively. For the TMM negative group, the survival prediction differed between classified *vs* associated cases, both when the CS lower level for association was 0.3, and when using the more stringent level of 0.5 (*P* 0.021 and 0.049, respectively).

#### Methylation classification in paired samples

**Fig. 6:**
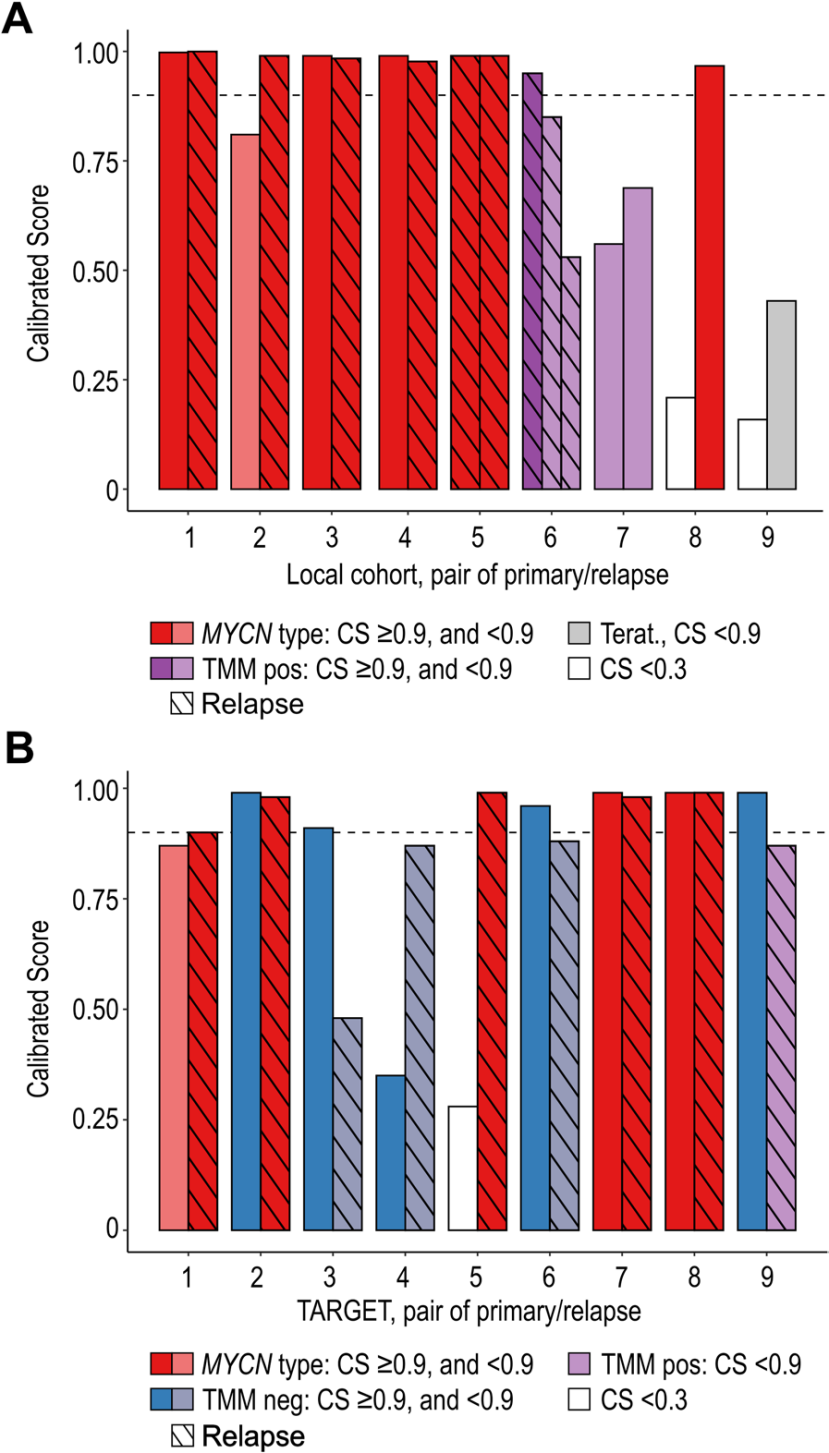
Methylation-based classification in paired tumour samples from same patient. **A** Nine paired tumour samples were evaluated in the local cohort. For three patients the primary tumour was analysed using both the 450 K and the EPIC array. For these pair 7 to 9, the 450 K-based classification is shown as left bar, and the EPIC-based classification is shown as the right bar. **B** Nine pairs of primary and relapsed tumours were evaluated in the TARGET cohort. Dotted lines show CS of 0.9. Terat., the molecular class “Teratoma (novel)”

Eighteen NB cases, nine in each cohort, were represented by more than one DNA methylation array, not including technical replicates. We verified paired patient identity through SNP clustering analysis (Supplementary Fig. 7) and compared the DNA methylation-based classification within pairs (Fig. 6). In the local cohort, no pair switched methylation classification. Four samples kept the classification (*MYCN* type, local pair 1, 3, 4, and 5), while the score increased from associated in the primary tumour to reaching classified in the relapse material in one case (*MYCN* type, pair 2). Pair 6 classified into TMM positive at first relapse but showed decreased CS in further relapse. Pair 7—9 were reanalysed (using new DNA preparations for pair 7 and 8) on the more comprehensive EPIC arrays. The classifications of these pairs increased in CS, but only pair 8 reached above CS 0.9 and received a *MYCN* classification. Contradictory to the assigned subclass, the case for pair 8 lacked support for MNA as judged from both SNP- and methylation array.

In the TARGET cohort, pairs from three cases classified both as primary and as relapse (pair 2, 7 and 8; Fig. 6B). Two of these pairs maintained a classification into the *MYCN* class, while the third sample switched from TMM negative class into *MYCN* type (pair 2). Neither of the primary tumours of pair 7 and 8 were reported to have MNA, which was also supported by low *MYCN* conumee-based estimates (Supplementary Table 3). Three pairs had an increased CS for the relapse sample (pair 1, 4, and 5; Fig. 6B), with relapse sample classification into *MYCN* type (pair 1 and 4) with a CS ≥ 0.9, despite a lower CS for the primary tumours. Primary samples of pair 1 and 4 were INSS stage 2b (discussed above) and stage 4, respectively. Three relapses moved from a TMM negative classification of the primary sample to a classification into *MYCN* type (pair 2: CS > 0.9), or an association with either *MYCN* type (pair 6: CS 0.88), or TMM positive (pair 9: CS 0.87) subclass, respectively. In no case, the classification, or association, switched into the TMM negative class, from another class. None of the paired samples had a major change in conumee estimated *MYCN* value (Supplementary Table 3), and an estimated *MYCN* value coherent with amplified *MYCN* was only found in pair 5, for both of its samples.

We next extracted the methylation-based CNA plots (Supplemental Figs. 8 and 9, which revealed that for several cases genomic events had occurred between the tumours specimens analysed at time of diagnosis and at time of relapse (Supplemental Tables 2 and 3). Interestingly, for local pair 3 and 4, as well as for TARGET pair 1, the divergent CNA plots suggest that relapse emerged either from a minor clone within the primary tumour or from an earlier common ancestral tumour cell (Supplementary Figs. 8 and 9). These three samples maintained the classification into MYCN type for the relapsed tumours.

## Discussion

Neuroblastoma is a heterogeneous disease, and patient stratifications are important for prognostication and to direct therapeutic interventions. For CNS tumours, DNA methylation-based classification has proven highly efficient in diagnosing and subgrouping tumours, and with its introduction into clinical diagnostics in several countries, we here sought to determine whether DNA methylation-based classification could be equally beneficial for neuroblastoma diagnostics. We therefore analysed tumours from 303 patients from two cohorts and correlated the findings with genomic information from the tumours and patient data.

At superfamily level, 94% and 86% of samples were classified correctly as neuroblastoma in the local cohort and TARGET, respectively, with 67% and 64% also subclassified (CS ≥ 0.9). As an optimal threshold for clinically relevant classifications, in terms of balance between sensitivity and specificity, has not been determined for the current classifier version (although 0.9 is recommended), we also assigned samples as associated for CS ≥ 0.3 but < 0.9. Our data do support that a CS below 0.9 could indicate an accurate molecular subclass, especially for the TMM positive samples, but the optimal threshold remains to be established. For the cases where fresh frozen material was used, the possibility for accurate estimation of the neoplastic content was limited. To circumvent inaccuracies due to low tumour content, samples presenting a flattened profile without distinct CNA were omitted from the analysis. Yet, variable tumour content in analysed samples could influence the obtained CS.

Almost all samples with confirmed MNA were classified to or associated with the *MYCN* type class with only two samples classified outside of the expected *MYCN* type. However, multiple cases showed classification or association to *MYCN* type without evident MNA (Fig. 3C). It should be noted that the classification does not take chromosomal copy numbers into account and only uses the methylation profile for classification. Thus, the samples classified as *MYCN* type are therefore expected to methylation-wise be quite similar regardless of MNA status and indicate that other genetical or biological factors can establish an epigenetically cell state otherwise associated with MNA. Interestingly, among the 12 cases in the local cohort that either associated or classified as *MYCN* type despite lack of MNA, six had an activating *ALK* mutation (data not shown), while one case had high-grade amplification of *CDK4* and *MDM2*. It has been shown that activated *ALK* can drive *MYCN* transcription [20] although, if this also results in a methylation pattern equivalent to *MYCN* type warrants further investigation. Similarly, one could speculate that *CDK4*/*MDM2* co-amplification activates proliferative pathways and introduces an epigenetic pattern that mimics the *MYCN* type. Kaplan–Meier analysis shows that patients in methylation class *MYCN* type and TMM positive have similar survival probability that is significantly poorer compared to TMM negative (Fig. 5). As MNA is a marker of high-risk disease and adverse outcome, it would be interesting to determine whether the patients classified in the *MYCN* type subgroup despite absence of MNA have comparable outcomes as MNA patients. The limited number of relevant patients, however, excluded us from performing this analysis.

Telomere retention in NB is commonly promoted by genomic alterations affecting *ATRX* or *TERT*, which were mutually exclusive events in the local cohort, in agreement with previous knowledge [10]. Telomere lengthening can also be promoted by MNA through *MYCN*-driven *TERT* re-expression, and interestingly, five occurrences of combined *TERT* alteration and MNA were noted in the local cohort (Fig. 2). These five cases retrieved highest score for *MYCN* type, which indicate that the MNA-associated methylation pattern may supersede, or overlap, with a TMM positive class driven by *TERT*. One case with a *TERT* aberration and 11q-deletion, but without MNA, was associated as TMM negative (CS 0.72). Although this is an unexpected methylation class in relation to the genomic finding, transcriptional data that could support a functional relevance of the specific *TERT* alteration through increased telomerase expression are unfortunately not available.

We had one occurrence with combined *ATRX* alteration and *MYCN* amplification (CS 0.68 and 0.29 for TMM positive and MYCN type, respectively, in the local cohort (Fig. 2). MNA and *ATRX* aberrations have been noted to be incompatible [21] and mutually exclusive in NB [10]. However, the limitations of the used method in combination with a possible presence of heterogeneous MNA, where neoplastic cells with and without MNA co-exist within the tumour [22], do not allow further specification on whether MNA and the *ATRX* aberration are present in the same cell population.

We further investigated chromosomal excess or loss that were representative for the subclasses using the conumee 2.0 algorithm, which showed that the three respective methylation classes have distinct copy number features. For the TMM positive class, enrichment of combinations of 11q loss, 3p loss, 7q gain, and 17q gain was noted, while a high proportion of *MYCN* type samples had a 1p deletion and 17q gain. These CNAs were previously known to be features of high-risk tumours [3]. Under the default setting the analysis tool was unfortunately too crude to clearly visualize the focal amplification of the *MYCN* gene, probably relating to large binning size in relation to size of *MYCN* (6.5 kb) [23] in combination with underestimation of relative gain in tumours with a modal karyotype above 2n. Thus, the graph indicated focal 2p amplification in a lower proportion of samples than anticipated. Regardless, the summary plots of TMM negative samples (Fig. 4, and Supplementary Fig. 4) indicate higher degree of copy number events consisting of whole chromosomal gains and losses than in the other subclasses, and the ploidy value of TMM negative samples was increased. This fits the current knowledge where whole chromosomal gain/loss is highly associated with good prognosis in neuroblastoma and thus, expected to lack TMM [3].

There are some limitations of our study. The cohort sizes may not encompass the full span of the biological heterogeneity of neuroblastoma. Most samples were primary tumours, and methylation-based classification of tumour evolution in paired primary and relapsed samples could only be investigated in a smaller subset of samples.

Our study indicates that if used in a clinical setting, one would anticipate that about two-thirds of analysed samples will obtain a score for a true match (CS ≥ 0.9) with a neuroblastoma subclass. An association (CS < 0.9) with TMM positive or *MYCN* type class seemed to contain supportive information on tumour biology associated with worse prognosis, and in line with this an association with TMM negative had a worse prognosis than those that classified (CS ≥ 0.9) into TMM negative subclass.

Detection of large genomic alterations such as MNA, 11q-deletion, other segmental and numerical alterations can be accurately inferred from the DNA methylation array data and could potentially replace a SNP array in this assessment. Although the resolution is limited for smaller focal CNA (e.g. intragenic *ATRX* deletions), the methylation-based classification can yet provide important information as we identified cases classified as TMM positive despite lacking evident genomic alteration associated with *TERT* or *ATRX*.

In summary, although the combination of genomic data and methylation-based classification showed that all cases classified as TMM positive (CS > 0.9) indeed presented features associated with TMM, the opposite association was not always true as several tumours positive for *TERT*- or *ATRX* aberration did not classify or associate as TMM positive. The genomic features of this group indicate that MNA, and possibly also *ALK* mutation, can induce a methylation pattern that supersede any pattern imposed by a *TERT* or *ATRX* aberration and recognized by the current version of classifier. However, as survival probabilities are equally poor for TMM positive and *MYCN* type and clearly separated from TMM negative, our study shows that DNA methylation-based classification can be used for diagnosing and subclassifying neuroblastoma into clinically relevant groups.

## Methods

### Dataset

#### Local cohort

Local cases were national/regional cases of NB collected between year 1986–2023 for which DNA methylation array data were available. Both primary and relapsed samples were included. Samples were quality checked using an in-house pipeline that builds on the minfi package with default settings. Samples with a probe fail rate ≥ 0.03 were not included in the study, and technical replicates from the same DNA preparation were omitted. Tumour cell content was unknown for these samples. Therefore, to ensure a high tumour cell content, we excluded samples that lacked distinct changes in copy number aberration profiles that had been present in the sample in other analyses (i.e. SNP microarray analysis). This step excluded 21 samples from the study, and the final dataset consisted of 90 NB subjects represented by 77 primary tumours, or 17 relapses tumours (status of primary/relapse was NA for two samples). There were 63 samples represented on the Illumina’s Infinium HumanMethylation450 BeadChip (450K, > 450 000 probes), the successor Infinium MethylationEPIC array (EPIC, > 850 000 probes) was used for 32 samples, and the further successor MethylationEPIC v2.0 array (EPICv2, > 930 000 probes) was used for 4 subjects. For 9 subjects, there was more than one methylation array available. Previously generated molecular data from SNP microarray and sequencing on *ALK*, *ATRX*, *MYCN* and *TERT* status, and C-circle assay [5] were used. Ethical permission was granted by the ethics committee (Karolinska Institutet and Karolinska University Hospital, registration number 22-07254-01, 2009/1369-31/1 and 03-736).

#### TARGET cohort

We also included the NB subjects with DNA methylation data from the comprehensive genomic profiled NB samples of the Therapeutically Applicable Research to Generate Effective Treatments (TARGET) initiative. These subjects had predominantly high-risk NB with stage 4, with the addition of some patients with stage 4 s. All samples had a high tumour cellularity (> 70%). NB samples of the TARGET initiative were accessed via the TARGET data matrix portal. Clinical data originated from the file TARGET_NBL_ClinicalData_Discovery_20221108.xlsx and were matched to DNA methylation arrays using the TARGET _NBL_samleMatrix_Discovery_20190808.xlsx, with file version two for both files. NB TARGET samples with both raw data of Infinium HumanMethylation450 BeadChip (Illumina) and available clinical information were used in our study. In total, there were 213 unique NB cases, and methylation array data from relapse were also available for 9 cases. One technical replicate from the same DNA preparation was omitted from further analyses.

In both the local and the TARGET cohort, the methylation array inborn SNP measurements were used to confirm that there was no sample mix-up among primary and relapse sample pairs (Suppl. Figure 7).

### DNA methylation-based classification of samples

The DNA methylation-based classification system for CNS tumours, version 12.5 (released in January 2022) and corresponding version 12.8 for EPICv2 at MNP www.molecularneuropathology.org/mnp/, which recently transferred to Epignostix was used to classify samples into methylation molecular classes (MC) and subclasses [24]. A sample was considered a match if the calibrated score (CS) was ≥ 0.9 to a Superfamily or to a molecular class/subclass, respectively. We use the terms “classify into” for CS ≥ 0.9, and “associate with” for CS < 0.9. Samples that received CS < 0.3 was considered to have no match.

### Copy number alteration analysis

Genomic copy number alterations (CNA) were estimated from DNA methylation array data using the R-package conumee 2.0 [23]. Simplified, this package uses the combined methylated and unmethylated signal for each probe to identify excess or loss of genetic material and uses normalized segmental chromosomal regions (approx.$\sim$ 15 000 bins for 450K arrays). A log2 ratio of normalized signals is presented over the chromosomes, where 0 represents the copy number neutral state. CNA was analysed for each sample, and summary genome plots were used to look at alterations in groups of samples. The function detailplot was used to obtain the conumee-based CNA estimate of the *MYCN* gene, using the default *MYCN* gene coordinates 16,080,683—16,087,129 bp at chromosome 2 (at genome build GRCh37/hg19).

### Statistical analysis

Statistical analyses were conducted in R version 4.3.1. Kaplan–Meier (K-M) curves for overall survival were generated using the function ggsurvplot of the R package survminer, using the time between date of diagnosis to last follow-up, or to death. For samples with relapses, the methylation class of the earliest available sample was used. Comparisons between groups was done using Mann–Whitney U Test. Log-rank test in the ggsurvplot function was used to compare K-M curves. *P* values of less than 0.05 were considered statistically significant.

## Supplementary Information

Additional file1 (PDF 8285 kb)

Additional file2 (XLSX 21 kb)

## Data Availability

The results published here are in part based upon data generated by the Therapeutically Applicable Research to Generate Effective Treatments (https://www.cancer.gov/ccg/research/genome-sequencing/target) initiative, phs000218. The data used for this analysis is available at the Genomic Data Commons (https://portal.gdc.cancer.gov). Other datasets used in the current study are available from the corresponding author on reasonable request.

## Abbreviations

NB: Neuroblastoma
TMM: Telomere Maintenance Mechanism
MNA: MYCN Amplification
ALT: Alternative Lengthening of Telomeres
TERT: Telomerase Reverse Transcriptase
ATRX: Alpha Thalassemia/Mental Retardation Syndrome X-Linked
DAXX: Death-Domain-Associated Protein
CNA: Copy Number Alteration
CS: Calibrated Score
MC: Methylation Class
SNP: Single Nucleotide Polymorphism
COG: Children’s Oncology Group
INSS: International Neuroblastoma Staging System
MYCN: MYCN Proto-oncogene, bHLH Transcription Factor
ALK: Anaplastic Lymphoma Kinase
MNP: Molecular Neuropathology Classifier
TARGET: Therapeutically Applicable Research to Generate Effective Treatments
IQR: Interquartile Range
