# Supplementary material for: DNA methylation profiling for molecular classification of neuroblastoma
Source: Clin Epigenetics. 2025 Jul 27;17:131. doi: 10.1186/s13148-025-01936-7 (PMC12297820; doi:10.1186/s13148-025-01936-7)
Supplement: Supplementary file 1 — Additional file1 (PDF 8285 kb) [file 13148_2025_1936_MOESM1_ESM.pdf]

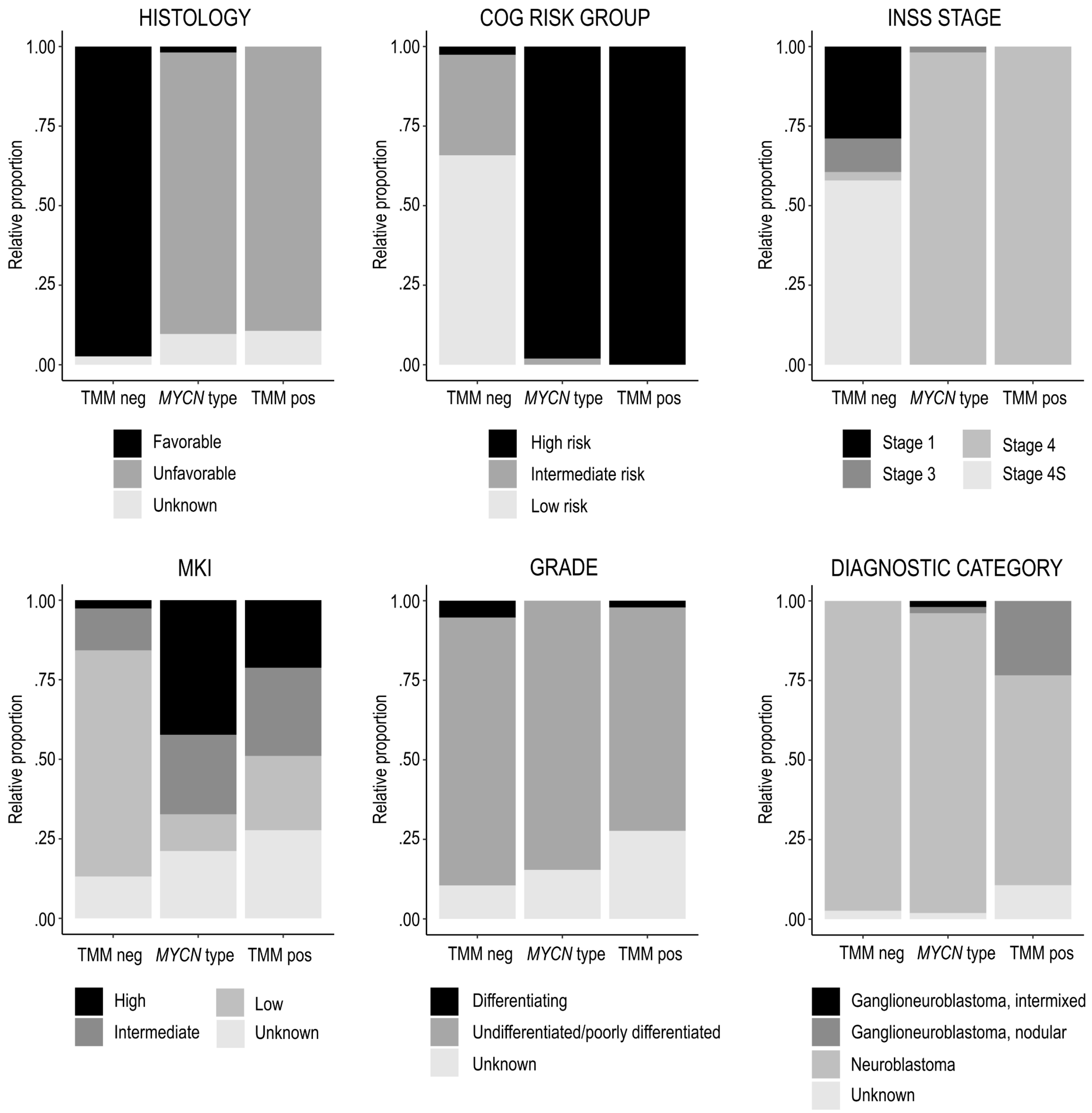

Supplemental Figure 1. Relative proportions of phenotypic variables in the TARGET cohort, associated with samples that classify with CS≥0.9. Number of patients in the subclasses are: $n_{TMM\ neg}$=38; $n_{MYCN\ type}$=52; and $n_{TMM\ pos}$=47. COG, Children's Oncology Group; INSS, International Neuroblastoma Staging System Stage; MKI, Mitosis-Karyorrhexis Index.

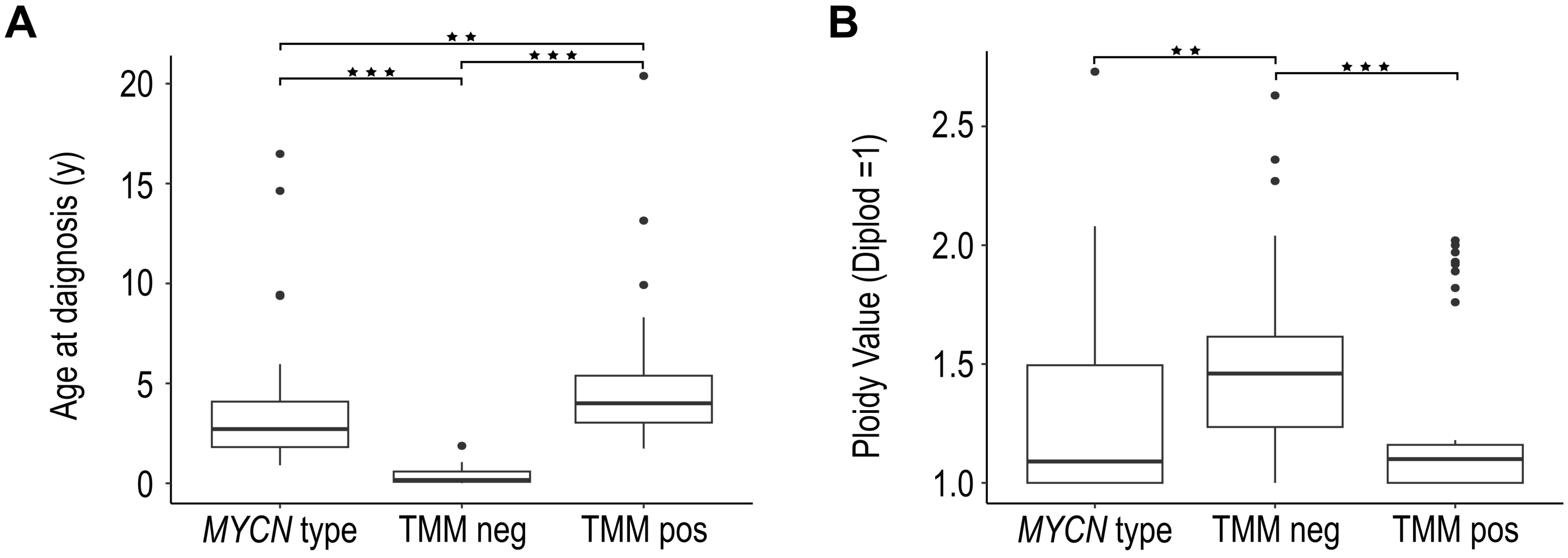

Supplemental Figure 2. Evaluation of phenotypic variables associated with samples that classify with CS≥0.9 in the TARGET cohort. Boxplots show **A.** the distribution of the variable age at diagnosis, and **B.** the distribution of ploidy value, of sample that classify into a subclass with a CS ≥0.9. A Ploidy value of 1 corresponds to the diploid genome. Number of patients in the subclasses are: $n_{\text{TMM neg}}$=38; $n_{\textit{MYCN}\text{ type}}$ =52; and $n_{\text{TMM pos}}$=47. ***, *P* value <0.001; **, *P* value <0.01

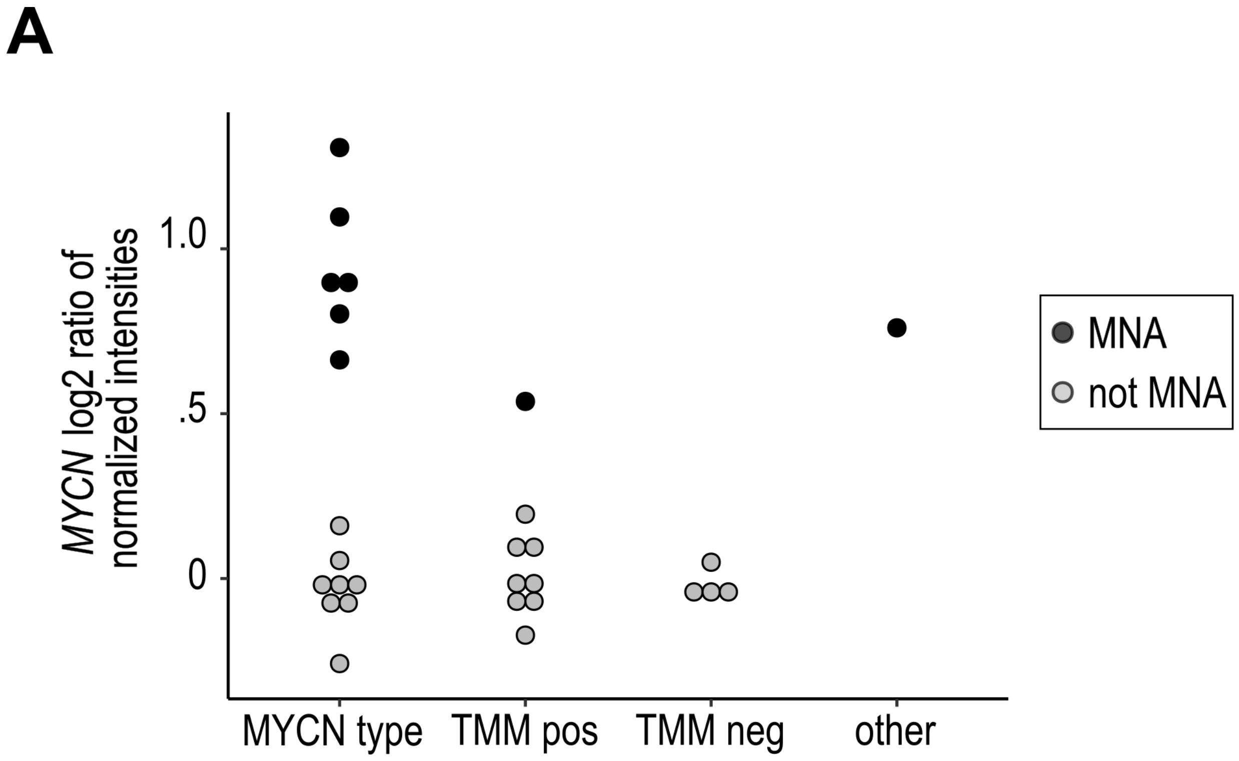

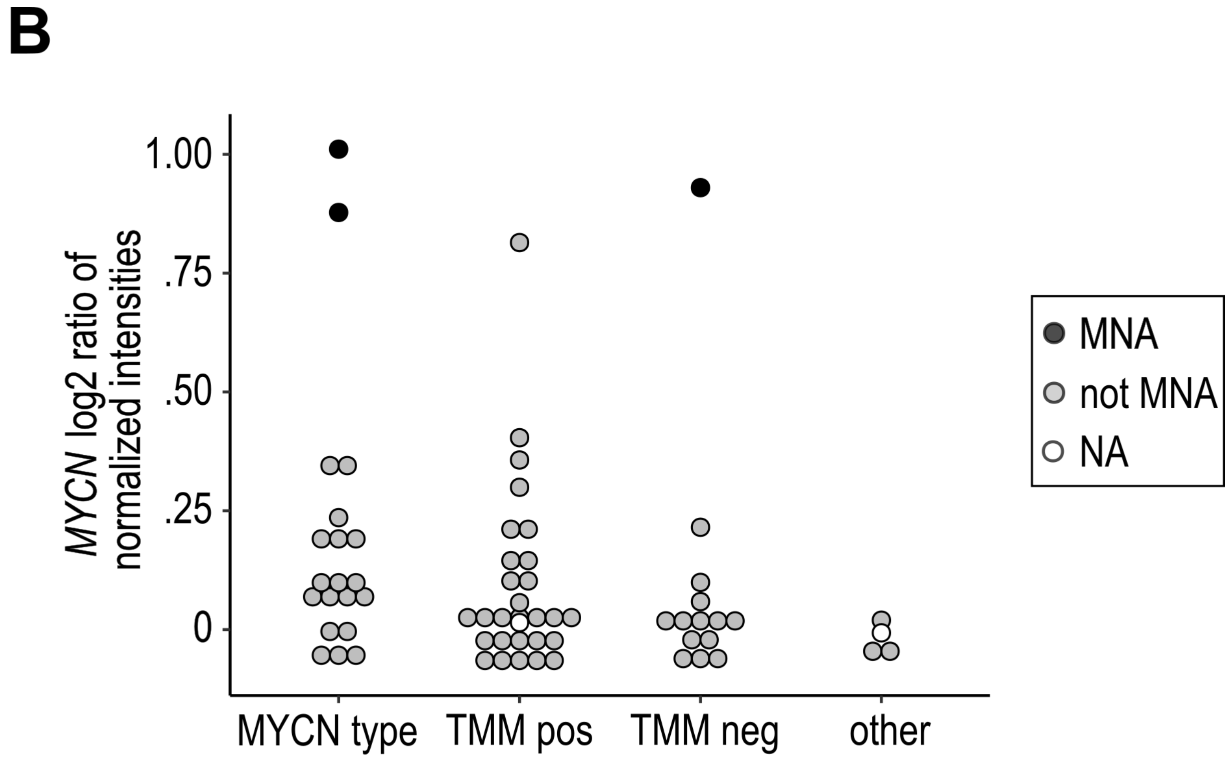

Supplemental Figure 3. Classification distribution of methylation-based MYCN-estimate in samples with CS <0.9. Tumours with methylation-based molecular classes of CS <0.9, and ≥0.3 are plotted against conumee-estimated relative *MYCN* amplification, in **A.** the local cohort, and **B.** in the TARGET cohort. In both cohorts, *MYCN* status was lacking for one tumour, respectively. MNA, *MYCN*-amplified; NA, not available.

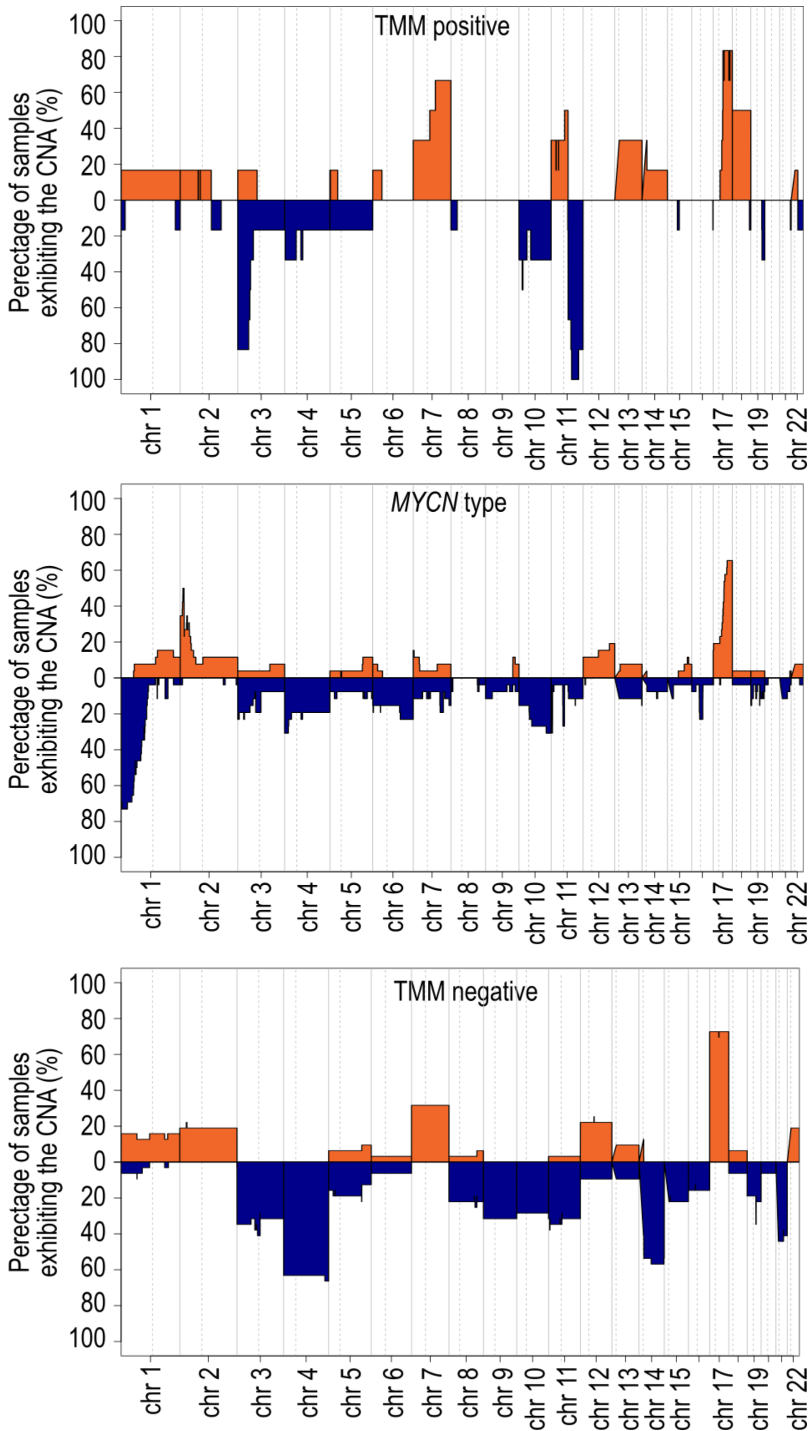

Supplemental Figure 4. Summary copy number prediction plots of neuroblastoma subclasses in local cohort. Percentage distribution of copy number alterations in samples that classified into TMM positive (n=7), MYCN type (n=26) and TMM negative (n=27) with CS ≥0.9 in the local cohort. Data from the EPICv2 array were omitted from the analysis (one TMM positive sample).

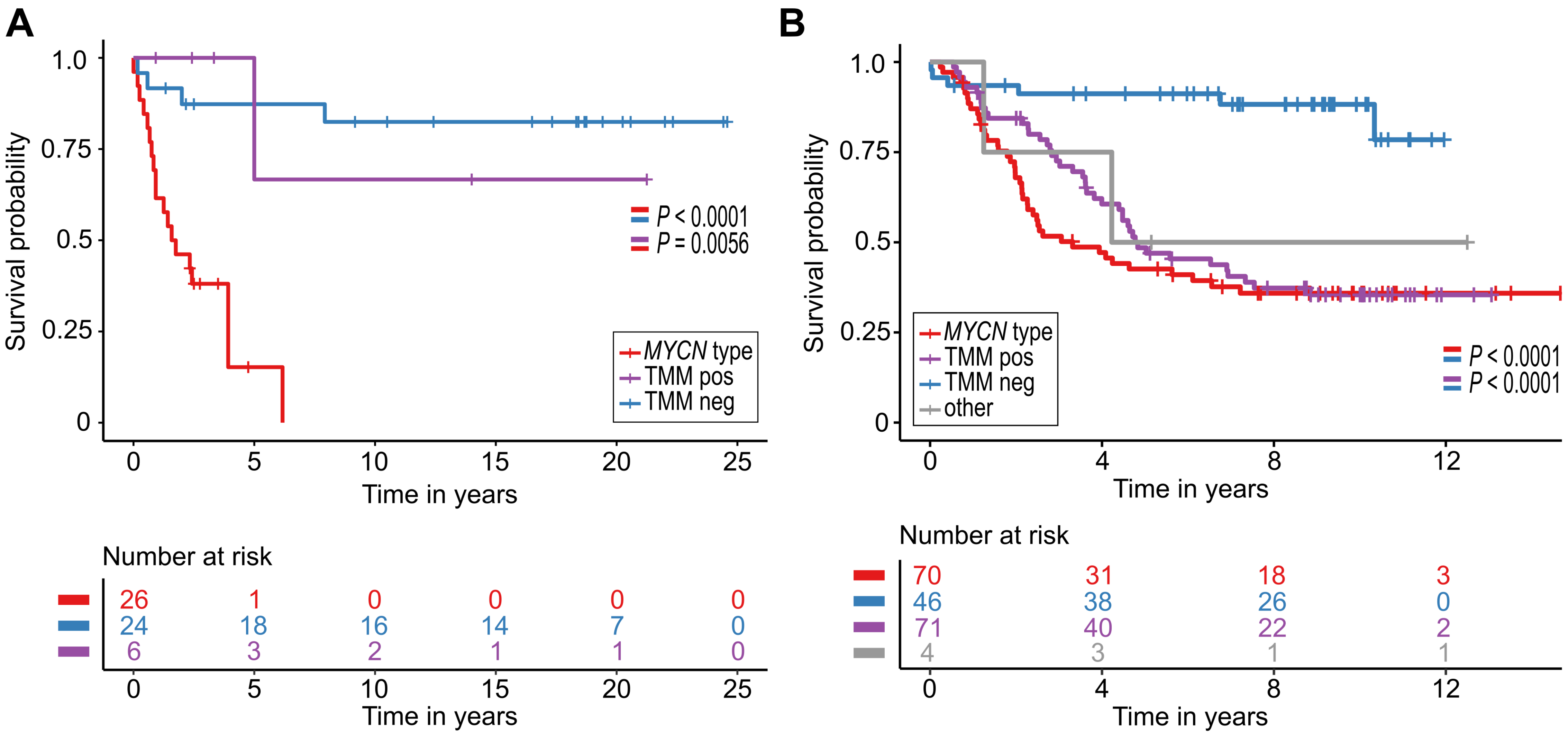

Supplemental Figure 5. Overall survival probability for primary tumours that associate or classify with CS ≥0.5. A. Kaplan-Meier plot show survival probability of the subclasses among 56 samples with a CS ≥0.5 in the local cohort. **B.** Kaplan-Meier plot show survival probability of the subclasses among 191 samples with a CS ≥0.5 in the TARGET cohort. The group "other" includes three samples that were associated with MC Schwannoma (n=3), and with MC Teratoma (n=1). Significant pairwise differences are shown in the plot.

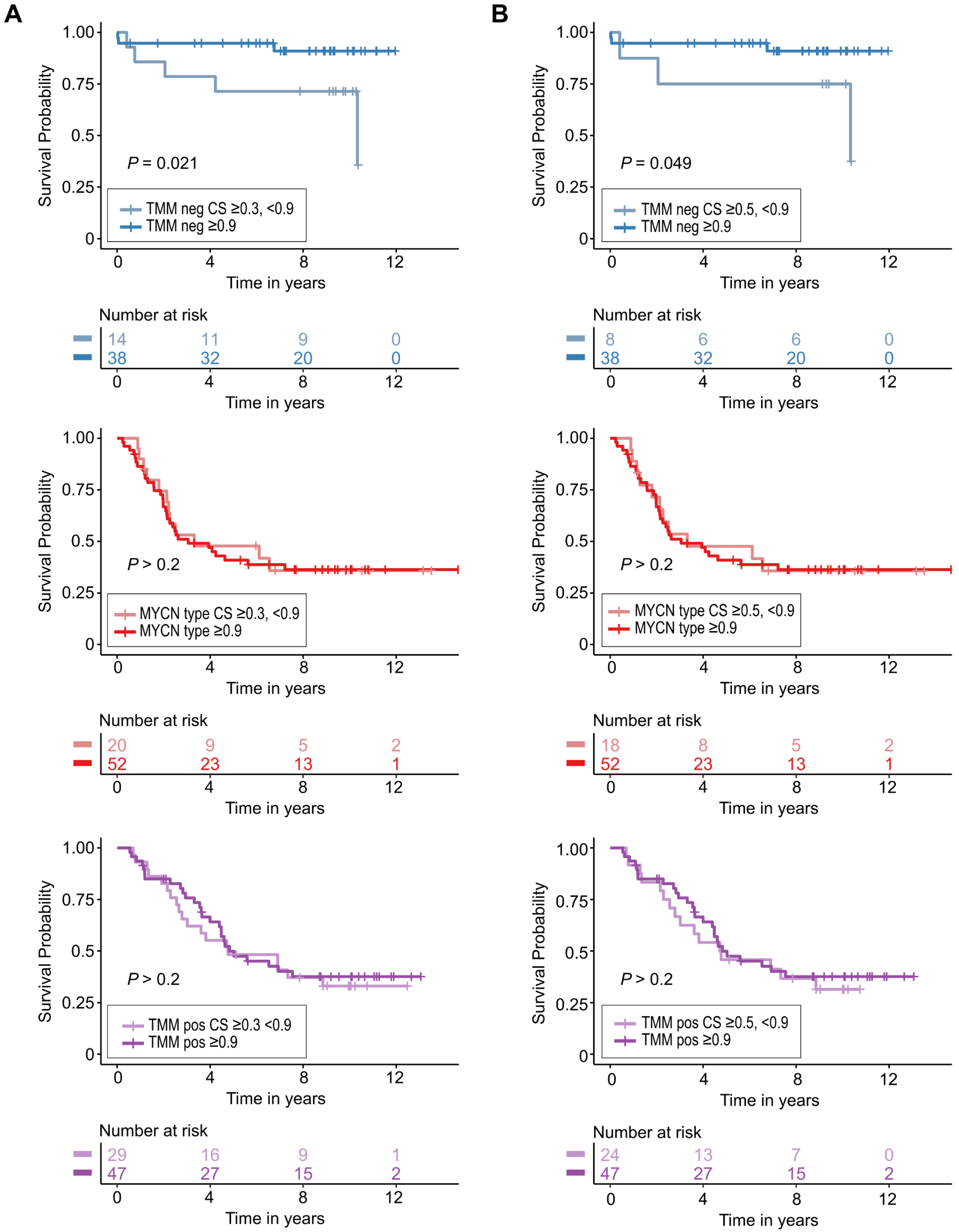

Supplemental Figure 6. Evaluation of impact of CS value on the overall survival probability for methylation-based classification of samples in the TARGET cohort. A. Kaplan-Meier plots from the survminer package show survival probability of the groups with CS ≥0.9 plotted against samples that associates with subclass at lower CS, for the subclasses TMM neg (top panel), *MYCN* type (middle panel), and TMM pos (lower panel). **A**. Samples in the group with the lower CS had a CS of <0.9, ≥0.3. **B**. Samples in the group with the lower CS had CS: <0.9, ≥0.5.

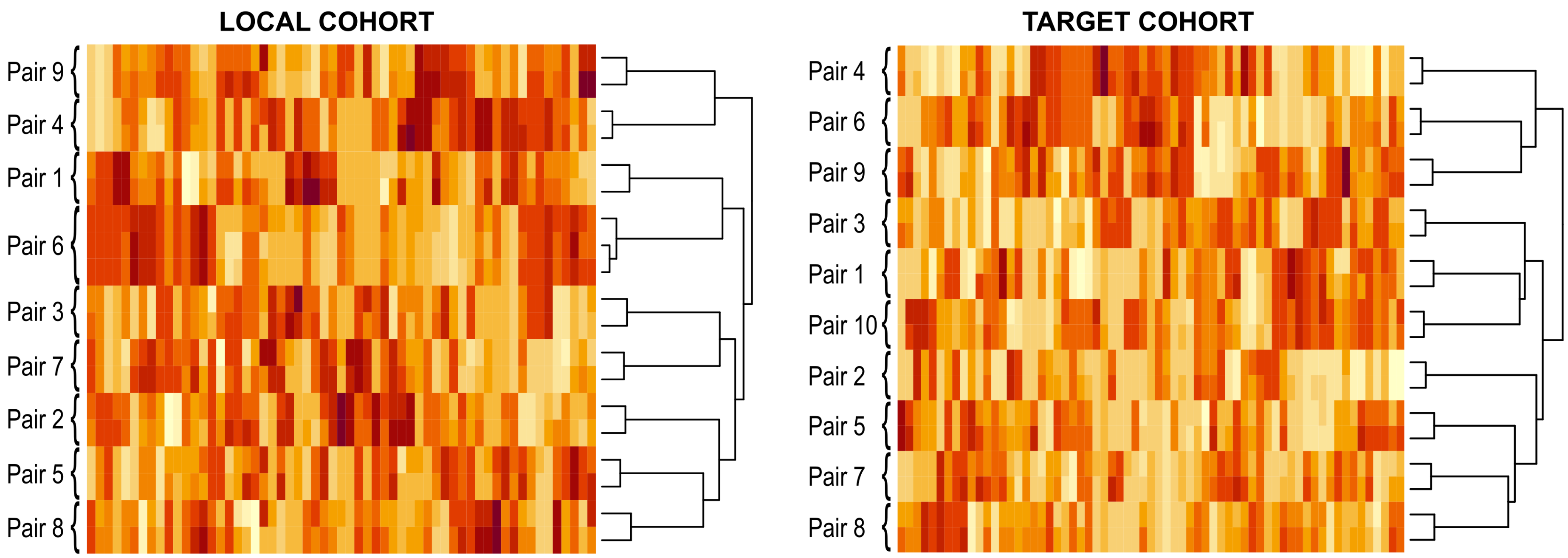

Supplemental Figure 7. Clustering of paired samples. Heatmap and dendogram of SNP data from the DNA methylation arrays show clustering of paired samples. **A**. 59 SNPs present on both 450K and EPIC arrays were used for clustering for 19 samples in the local cohort. **B.** 65 SNPs present on 450K arrays were used for clustering of 20 paired samples in the TARGET cohort, with the 10th pair representing a technical duplicate.

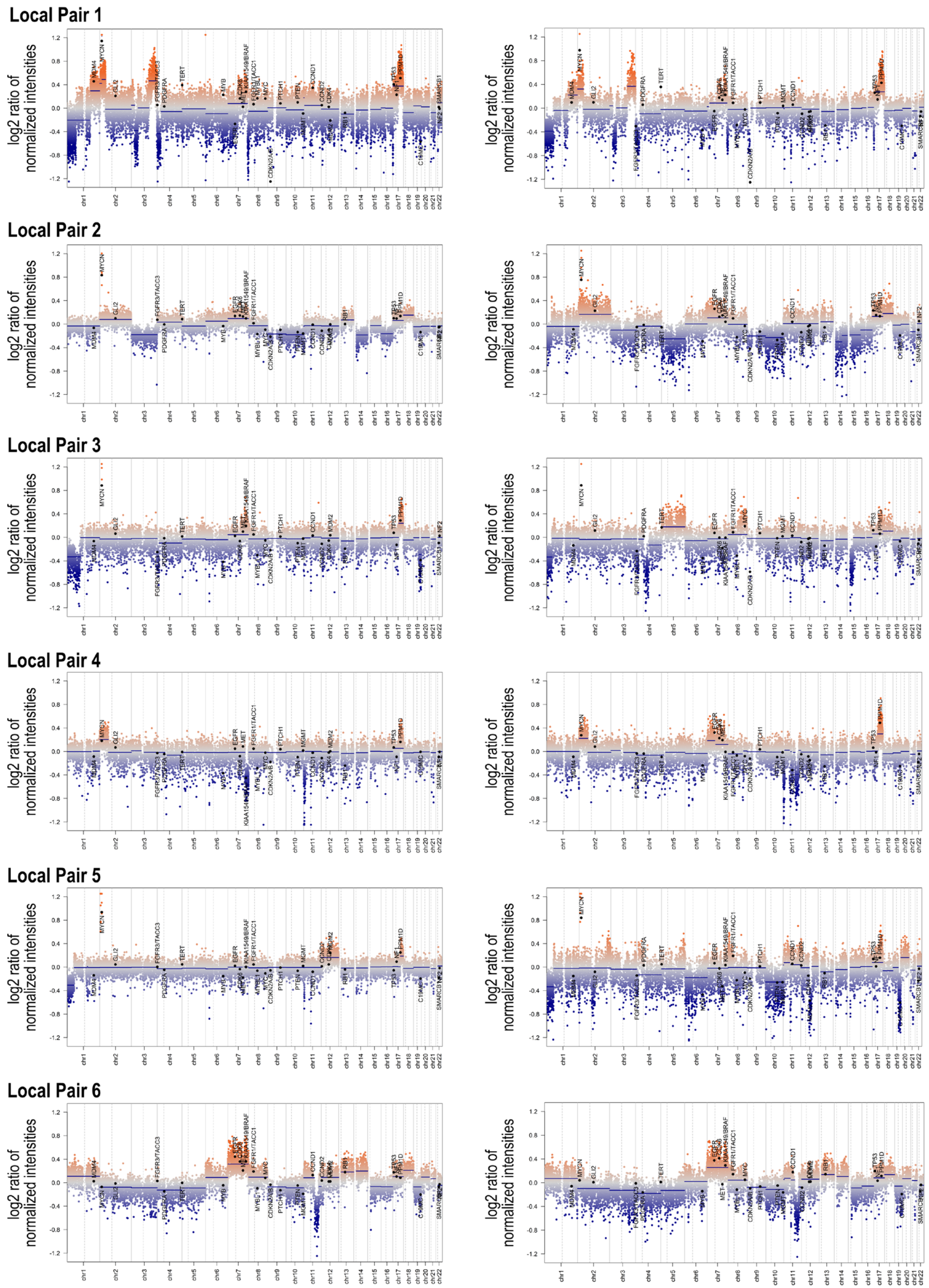

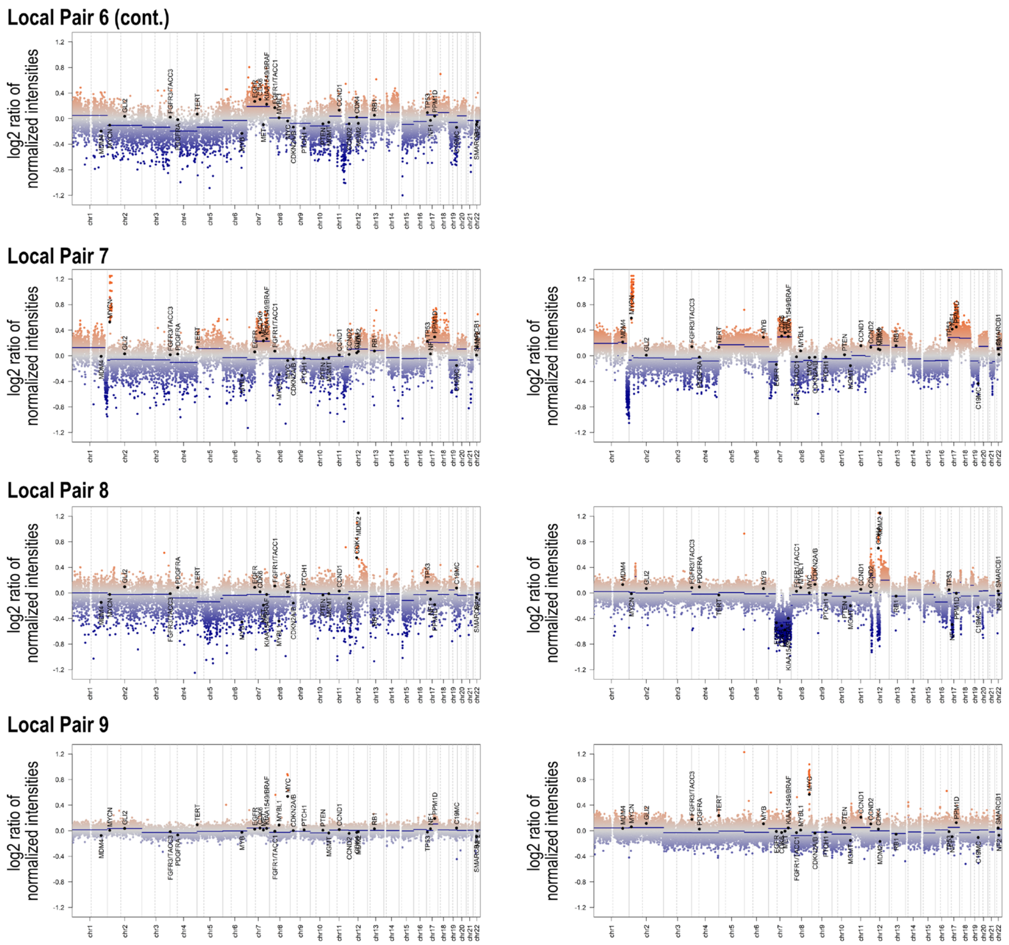

Supplemental Figure 8. Copy Number Prediction plots of paired samples in the Local cohort. Samples appear in the same order as in Figure 6, within each sample set. In sample 1-4 pair comprises of primary and relapsed sample. In sample 5 and 6, all samples are relapses. For pair 7 - 9 both samples within pairs are from primary tumour, displaying data from 450K array (left panel), and data from EPIC array (right panel).

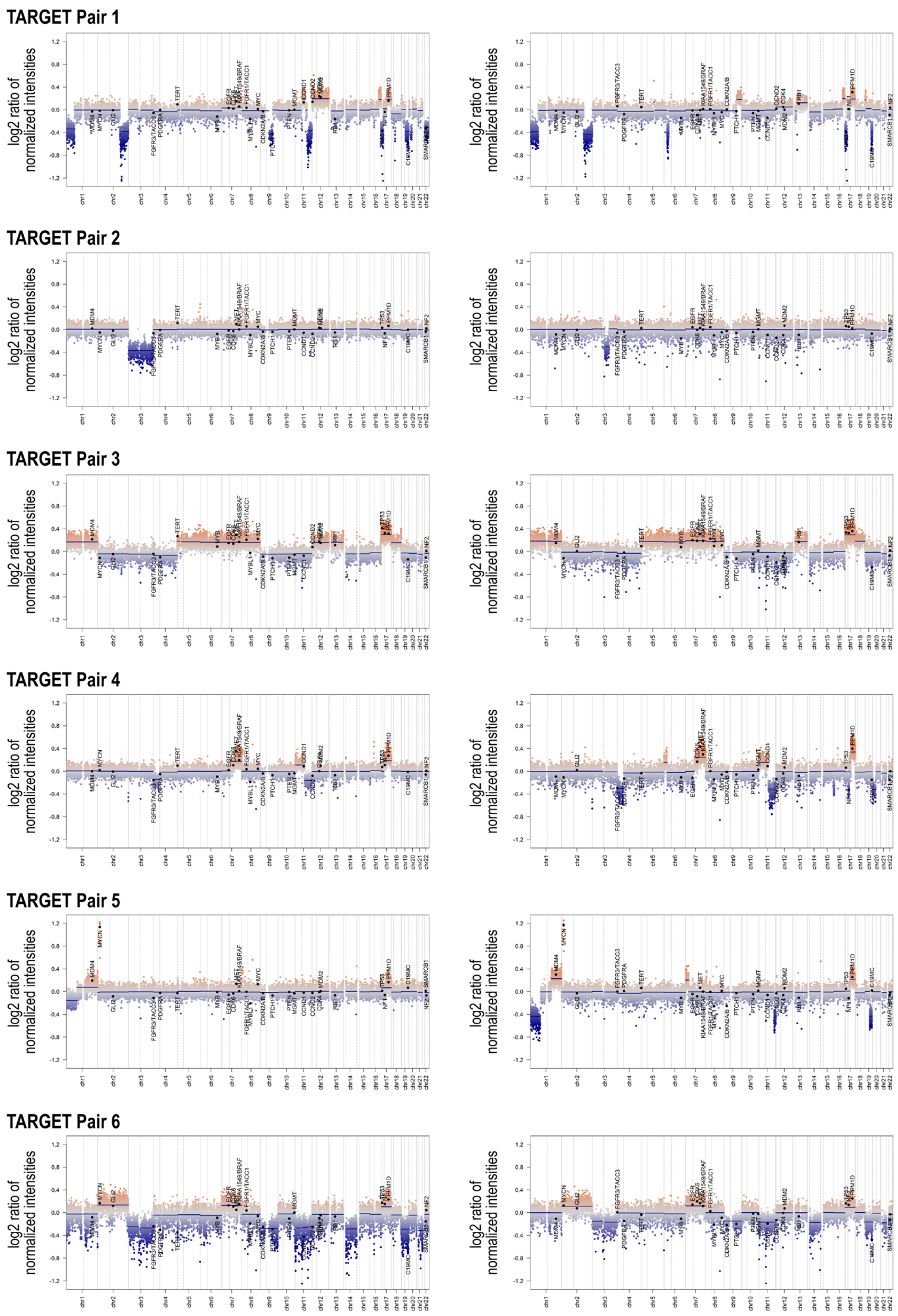

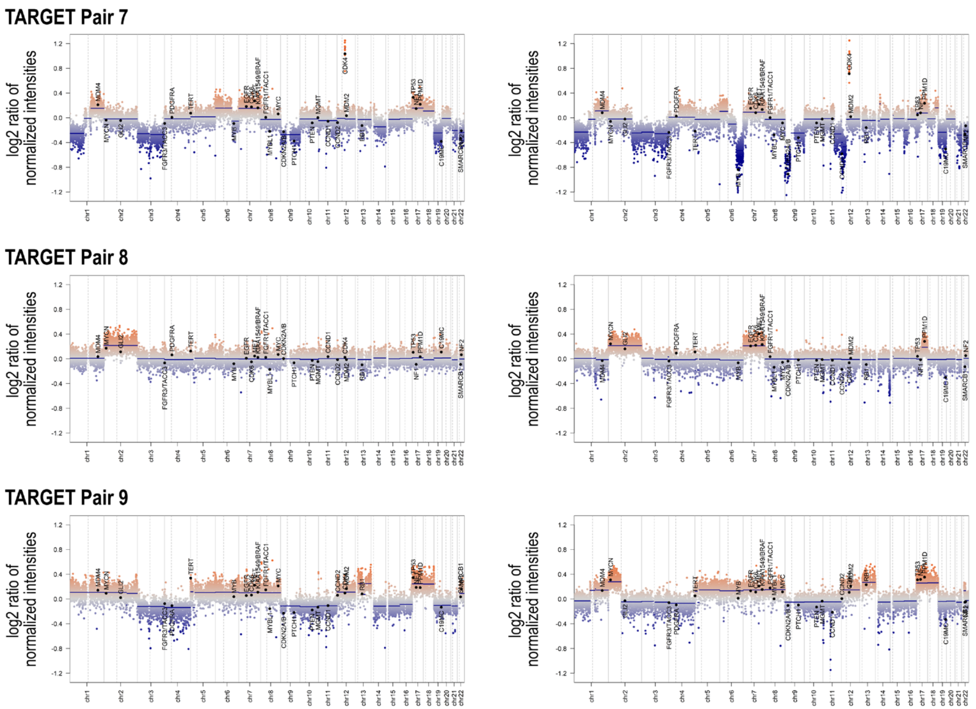

Supplemental Figure 9. Copy Number Prediction plots of paired samples in the local cohort. Samples appear in the same order as in Figure 6, within each pair and graphs show primary sample (left panel) and relapse (right panel).
